# Multicomponent reaction access to complex quinolines via oxidation of the Povarov adducts

**DOI:** 10.3762/bjoc.7.110

**Published:** 2011-07-13

**Authors:** Esther Vicente-García, Rosario Ramón, Sara Preciado, Rodolfo Lavilla

**Affiliations:** 1 Barcelona Science Park, Baldiri Reixac 10–12, 08028, Barcelona, Spain; 2 Laboratory of Organic Chemistry, Faculty of Pharmacy, University of Barcelona, Avda. Joan XXIII sn, 08028, Barcelona, Spain

**Keywords:** manganese dioxide, multicomponent reactions, oxidation, Povarov, quinolines, tetrahydroquinolines

## Abstract

The tetrahydroquinolines obtained through the Povarov multicomponent reaction have been oxidized to the corresponding quinoline, giving access to a single product through a two-step sequence. Several oxidizing agents were studied and manganese dioxide proved to be the reagent of choice, affording higher yields, cleaner reactions and practical protocols.

## Introduction

**Scheme 1:**
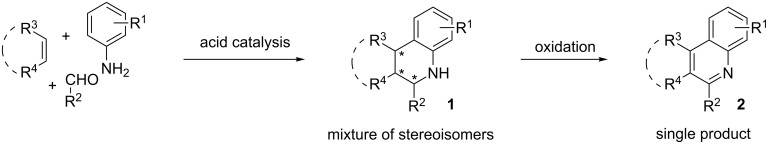
Povarov oxidation access to substituted quinolines.

Heterocycles are ubiquitous scaffolds in pharmaceuticals, natural products and biologically active compounds. Quinoline systems in particular constitute a privileged substructure and are present in a large number of compounds with remarkable biological activity [1]. Although a variety of methods are used to prepare these heterocyclic compounds, the synthetic access to polysubstituted-polyfunctionalized derivatives remains a serious challenge [2]. Multistep sequences are widespread in the literature, but even in these cases the preparation of some substitution patterns and functional group combinations is particularly difficult. The recent introduction of multicomponent reactions (MCRs) into this field has brought interesting features typical of the ideal reaction, such as atom- and step economy, convergence, and exploratory power, together with new avenues in connectivity, leading to the straightforward synthesis of previously unobtainable scaffolds [3]. In this context, it is possible to obtain a wide variety of complex tetrahydroquinolines through the Povarov MCR (the interaction of anilines, aldehydes and activated olefin inputs under acid catalysis) [4–8]. Interestingly, this process allows cyclic enol ethers and enamines to be used as electron-rich alkenes, leading to heterocycle-fused tetrahydroquinolines, usually as a mixture of stereoisomers [9–13]. Unfortunately, no general methods for enantioselective Povarov reactions have been developed (for examples of catalytic enantioselective transformations operating in particular systems, see [14–15]), and this constitutes a serious drawback in the use of this reaction for library preparation, as one reaction affords several products, when ideally it should give only one. However, these adducts can be subjected to oxidation, which will lead to the corresponding quinolines, preserving the substituents and functionalization already introduced in the preceding MCR. Despite the loss of all stereochemical information, in this way it would be feasible to obtain a single product from a multicomponent process (Scheme 1).

The oxidation step itself is challenging as it involves the formal removal of four hydrogens from a tetrahydropyridine moiety to reach the fully aromatic species. The literature contains scattered reports of the use of oxidants for this transformation: 2,3-Dichloro-5,6-dicyano-1,4-benzoquinone (DDQ), ceric ammonium nitrate (CAN), nitrobenzene, elemental sulfur, palladium and manganese dioxide among others, all of them far from being ideally suited for these substrates.

**Scheme 2:**
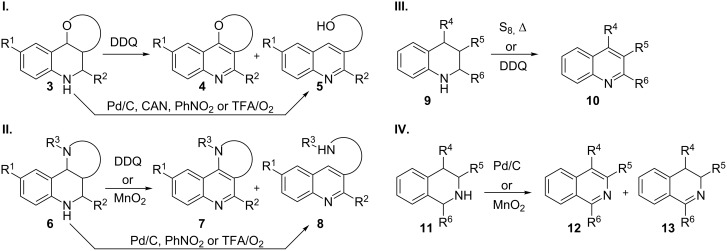
Tetrahydroquinoline oxidation.

One of the most commonly used is DDQ, which affords quinolines in acceptable yields. The main advantages of this oxidizing agent lie in its chemoselectivity and a requirement for relatively mild conditions, allowing it to be used in the presence of a wide range of substituents of the starting tetrahydroquinoline, such as O-, N- and C-linked residues (Scheme 2) [8–9,12–13,16–18]. Unfortunately, the alternative oxidation–elimination products (**5** and **8**) are often observed, therefore suggesting an acid catalyzed process. This would account for the elimination of alcohol and amine moieties, leading to dihydroquinoline intermediates that, after spontaneous oxidation in air, provide the final fragmented quinolines. The ability of DDQ to act as a Lewis acid and promote this alternative pathway has some precedent in the literature [19]. Furthermore, TFA treatment of Povarov adducts in oxygenated atmospheres also affords the oxidation–elimination products **5** and **8** (Scheme 2) [8,12,20].

The alternative oxidation–elimination pathway is predominant in some CAN-promoted oxidations of different Povarov adducts **3**. Incidentally, this reagent is also used as a catalyst in the Povarov MCR without oxidative interference [18]. The same trend (oxidation–fragmentation) can be observed using nitrobenzene [21] as the oxidant. Analogously, elemental sulfur and palladium, although requiring drastic conditions, also lead to the fragmented quinolines when the substrates bear O- and N-substituents [22–25] (for related isoquinoline oxidations, see [26–27]).

Related oxidative processes involve, for instance, a cascade Povarov–hydrogen transfer reaction using Tf_2_NH as a catalyst and the imine as an oxidant, as recently described [28]. In addition, Povarov adducts resulting from the reaction between 3-aminocoumarin, aldehydes and cyclic enol ethers have been oxidized with different types of reagents, such as bromide, palladium, DDQ, sodium periodate, manganese dioxide or CAN, but in all cases the main product was the elimination–oxidation compound [29].

Finally, chemical manganese dioxide (CMD) has been widely used in this type of transformations, and already in 1982 the oxidation of tetrahydroisoquinoline (**11**, Scheme 2) was reported to yield the corresponding isoquinoline **12**, the intermediate dihydroisoquinoline **13** being obtained as a by-product [26]. Later, Thompson et al. described the oxidation of fused pyrrolohydroquinolines (type **6**) using MnO_2_ obtained from batteries. A kinetic competition between two processes was observed, and the desired double oxidation to the corresponding fused quinoline **7** took place, along with the oxidation–elimination sequence leading to **8**. A large excess of oxidant was required in order to obtain the desired quinoline **7** as the major product (Scheme 2) [30–31].

## Results and Discussion

Experiments were performed with the goal of developing a general and practical protocol for the oxidation of Povarov adducts to furnish the corresponding fused quinolines, avoiding elimination by-products. After unsuccessful attempts using palladium on carbon (decomposition), CuCl (partial oxidative elimination), Fremy’s salt (unreactive) and IBX (a complex reaction leading to unknown compounds), we focused our attention on MnO_2_ as the oxidant of choice. A literature search revealed different reactivity patterns depending on the type and origin of the reagent, with the commercial source being particularly important [32–36]. A systematic study was therefore conducted to determine the influence of different reaction conditions, commercial reagents and additives on the oxidation of an elimination-prone Povarov tetrahydroquinoline substrate.

**Scheme 3:**
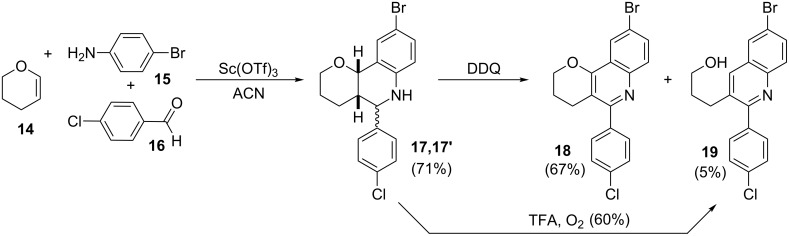
Synthesis of the Povarov adducts and their oxidation products.

In this way, tetrahydroquinolines **17**,**17'** were synthesized as a mixture of isomers from the enol ether **14**, *p*-bromoaniline (**15**) and *p*-chlorobenzaldehyde (**16**) under Sc(OTf)_3_ catalysis using standard reaction conditions (Scheme 3) [9]. Subsequently, these adducts **17**,**17'** were oxidized with DDQ by the standard protocol [9], to isolate the desired quinoline **18** and its fragmented derivative **19**, and they could also be subjected to an acid treatment to obtain selectively the latter product [8]. All compounds were purified and unequivocally characterized by NMR and HPLC methods.

Taking into account that the oxidation of thiazolidines to thiazoles with MnO_2_ (25 equiv) in toluene (55 °C) in the presence of pyridine (1.25 equiv) is a clean and efficient method [35], a first experiment was set up to test these conditions with an old (≈40 years) MnO_2_ sample of unknown origin (particle size 11.46 µm, see below). A promising result was obtained, achieving a 39% conversion to the desired product **18**, albeit with a high ratio of the elimination–oxidation compound **19**. Next, the equivalents of oxidant and pyridine were increased to 100 and 6, respectively, and under these optimized conditions, a 72% isolated yield of quinoline **18** was obtained, and no starting material or elimination–oxidation compound was detected.

Unfortunately, we were not able to reproduce the above results when using brand new samples of MnO_2_. It was decided to test different commercially available MnO_2_ sources (Aldrich, Acros and Wako) of distinct activation degrees (particle size, powder or activated reagent, Table 1) in order to find a suitable reagent leading to comparable results.

**Table 1:** Survey of different MnO_2_ reagents.

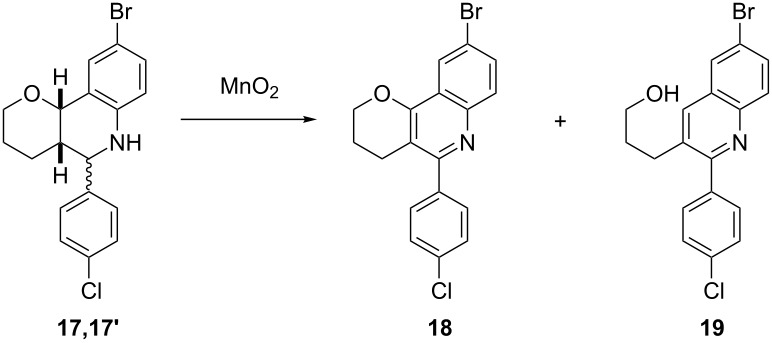				

entry	MnO_2_ trademark, characteristics (reagent code)	particle size (median diameter, d_50_, µm)^a^	reaction conditions	product ratios (**17**,**17'**)/**18**/**19**

1	Aldrich, reagent grade (310700)	4.3	25 equiv of oxidant	54/3/43
2	Aldrich, reagent grade (310700)	4.3	pyridine (50 equiv)	48/8/44
3	Aldrich, reagent grade (310700)	4.3	25 equiv of oxidant K_2_CO_3_ (6 equiv)	37/6/57
4	Aldrich, reagent grade (310700)	4.3	55 °C for 14 h	37/13/50
5	Aldrich, reagent grade (310700)	4.3	rt for 48 h	51/0/49
6	Aldrich, reagent plus (243442)	138.4	general conditions^b^	100/0/0
7	Aldrich, reagent plus (243442)	138.4	110 °C for 14 h	61/0/39
8	Aldrich, activated (217646)	4.2	general conditions^b^	8/14/78
9	Acros, powder (213490010)	7.6	general conditions^b^	75/0/25
10	Wako, 1^st^ grade powder (138-09675)	25.7	general conditions^b^	0/100/0

^a^All manganese dioxide samples were analyzed with a LS^TM^ 13 320 series Laser diffraction particle size analyzer. For more details, see Supporting Information File 1. ^b^Unless otherwise stated, the reactions were performed in toluene as the solvent, using 100 equiv of oxidant, 6 equiv of pyridine at 55 °C for 2 h.

Aldrich MnO_2_ (reagent grade) did not afford the desired quinoline **18** (entry 1, Table 1), the main products being the fragmented quinoline **19** and starting material. Modifications including the use of a greater excess of pyridine, the addition of K_2_CO_3_ as a heterogeneous base (entries 2 and 3), and adjustment of the reaction time or temperature (entries 4 and 5) did not substantially change the outcome. MnO_2_ (Aldrich, reagent plus) was completely inefficient at 55 °C (entry 6), and on heating to 110 °C for 14 h it promoted a 39% conversion but led exclusively to the elimination product (entry 7). On the other hand, using activated MnO_2_ (Aldrich), some oxidized quinoline **18** was observed, although again the predominant product was the fragmentation compound **19** (entry 8). Next, the reagents from Acros (entry 9) and Wako (entry 10) were tested, the latter being selective in the formation of the desired oxidation product, completely avoiding the elimination pathway. The results were reproducible, allowing the isolation of quinoline **18** in 66% yield in gram scale quantities.

In an attempt to improve the reaction conditions, Et_3_N was tested as a base, and molecular sieves (4 Å) and MgSO_4_ were introduced as dehydrating agents, but no meaningful changes were observed in any case. As the elimination–oxidation product **19** is thought to be generated by the acid characteristics of the oxidation reagents, an activated MnO_2_ sample was treated with an aqueous basic (NaCO_3_) solution, in an attempt to neutralize the acidic impurities, but the ratio of the elimination–oxidation product did not decrease. We then analysed the particle size of all samples using a laser diffraction technique (see Supporting Information File 1). Although a straightforward conclusion is not evident, it seems that all samples with a small (around 4 µm) or large particle size (138 µm) were inefficient in promoting the desired oxidation. On the other hand, medium size samples (Wako and the old sample of unknown origin) were the most selective oxidants (see Supporting Information File 1).

**Figure 1:**
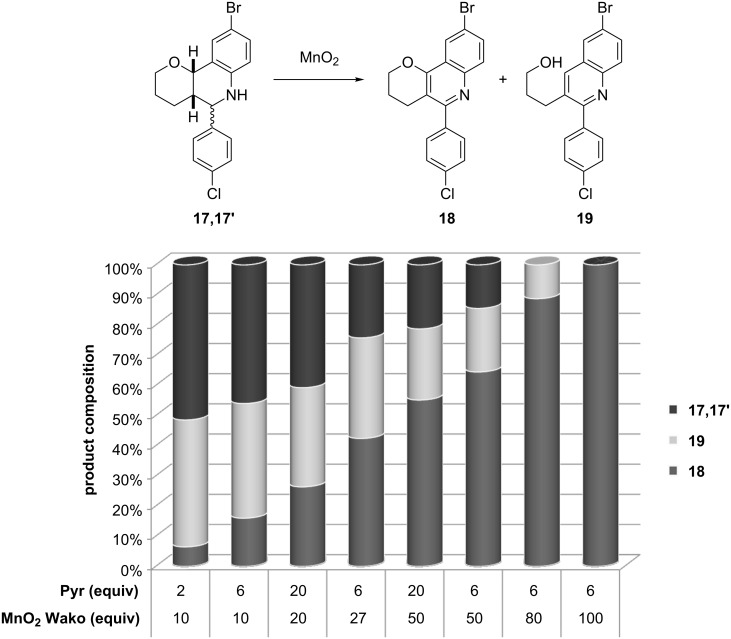
Optimization of the reaction conditions for the preparation of quinoline **18**.

Using this reliable reagent, different reaction conditions were tested in order to optimize the process, especially regarding reagent consumption (Figure 1). The effects of varying the amounts of Wako MnO_2_ (from 10 to 100 equiv) and pyridine (from 2 to 20 equiv) in the standard solvent (toluene), reaction time and temperature (2 h, 55 °C) were studied. The gradual increment in the amount of oxidant resulted in a progressive increase in the yield of compound **18** and the simultaneous decrease of the elimination quinoline **19**. No productive transformation to quinoline **18** was observed using 10 equiv of oxidant, the fragmented compound **19** being the predominant species. It is worth noting that the conversion of the starting material was only complete when at least 80 equiv of MnO_2_ were used, but even in these conditions the elimination pathway could not be completely avoided, despite the huge excess of pyridine (up to 20 equiv). As such large amounts of pyridine were not beneficial, the use of 6 equiv of this reagent was a practical compromize, leading to the same essential outcome. In an attempt to disaggregate the Wako MnO_2_ powder, and in this way reduce the amount of reagent, the reaction was performed in an ultrasonic bath under the general conditions, but no improvement was observed in the reaction profile.

**Scheme 4:**
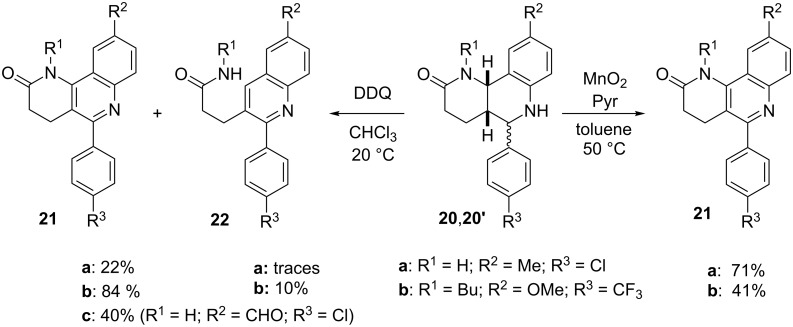
Oxidation of lactam-fused tetrahydroquinolines **20**,**20'**.

The optimized oxidation conditions were applied to another class of tetrahydroquinolines, which contain a fused lactam ring (**20**,**20'**, Scheme 4) [12]. These new substrates were prepared through the Povarov MCR from the corresponding unsaturated lactam, aldehyde and aniline. The oxidation and elimination products (**21** and **22**, respectively) were independently prepared with DDQ under acid catalysis in an oxygenated atmosphere (O_2_-TFA), and characterized by NMR and HPLC methods. The optimized conditions with the Wako reagent were productive and selectively afforded the corresponding quinolines **21** in high yields, and the elimination product **22** was not detected. The processes were slower (5–8 h) than those involving the pyran-fused substrates **17**,**17'** (Scheme 3). Interestingly, although DDQ is also capable of promoting these transformations, it is not as selective as Wako MnO_2_, and apart from yielding the fragmented quinolines **22**, it also oxidizes the benzylic hydrogens (**a** series, R^2^ = Me) leading to the corresponding aldehyde derivative **21c** [12]. Studies are ongoing to expand this set of transformations to fused oxygenated and nitrogenated 5-membered ring systems.

## Conclusion

In conclusion, we have described a fast, practical and reliable methodology to oxidize complex polysubstituted tetrahydroquinolines, arising from Povarov MCRs, to the corresponding quinolines, using MnO_2_. The influence of the reagent source, stoichiometry, additives and reaction conditions has been determined. Wako CMD is the oxidant of choice and the presence of pyridine is critical to avoid the fragmentation pathway, a side reaction often found in this type of transformation. This process enables the selective preparation of heterocycle-fused quinolines arising from a single combination of aldehydes, anilines and activated alkenes in a short sequence, involving Povarov MCR and oxidation steps.

## Experimental

### General

^1^H and ^13^C NMR spectra were recorded on a Varian Mercury 400 spectrometer. Unless otherwise stated, NMR spectra were recorded in CDCl_3_ solution with TMS as an internal reference. Data for ^1^H NMR spectra are reported as follows: Chemical shift (δ ppm), multiplicity, integration and coupling constants (Hz). Data for ^13^C NMR spectra are reported in terms of chemical shift (δ ppm). Signals were assigned by means of two-dimensional NMR spectroscopy: ^1^H,^1^H-COSY, ^1^H,^13^C-COSY (HSQC: heteronuclear single quantum coherence) and long-range ^1^H,^13^C-COSY (HMBC: heteronuclear multiple bond connectivity). IR spectra were recorded using a Thermo Nicolet Nexus spectrometer and are reported in wavenumbers (cm^−1^). High resolution mass spectrometry was performed by the University of Barcelona Mass Spectrometry Service.

#### General procedure A [9,12]

To a solution of compound **17**,**17'** or **20**,**20'** (1 mmol) in 15 mL of CHCl_3_, DDQ (2 mmol) was added and the mixture was stirred for 24 h in an open vessel at room temperature. An aqueous saturated NaHCO_3_ solution (10 mL) was added, and the resulting mixture was extracted with CHCl_3_ (3 × 10 mL). The combined organic layers were dried over Na_2_SO_4_, filtered and concentrated in vacuo. The reaction mixture was purified by flash chromatography (hexane–EtOAc) to afford the desired product.

#### General procedure B [9,12]

To a solution of compound **17**,**17'** or **20**,**20'** (1 mmol) in CH_3_CN/H_2_O or CHCl_3_/H_2_O (1:1, 6 mL), TFA (2 mmol) was added. The reaction mixture was stirred for 24 h at room temperature, quenched with an aqueous saturated NaHCO_3_ solution (10 mL) and extracted with CH_2_Cl_2_ (3 × 10 mL). The combined organic layers were dried over Na_2_SO_4_, filtered and concentrated in vacuo to give a residue which was purified by flash chromatography (hexane–ethyl acetate) to afford the desired product.

#### General procedure C

To a solution of compound **17**,**17'** or **20**,**20'** (1 mmol) in 50 mL of toluene, pyridine (6 mmol) and MnO_2_ Wako (100 mmol) were added and the mixture was stirred in an open vessel at 55 °C. The progress of the reaction was controlled by TLC or HPLC, until the starting material completely disappeared or no evolution was observed. The crude mixture was filtered through Celite, and the filtrate was concentrated in vacuo. The reaction mixture was purified by flash chromatography (hexane–EtOAc) to afford the desired product.

#### 9-bromo-5-(4-chlorophenyl)-3,4-dihydro-2*H*-pyrano[3,2-*c*]quinoline (18)

Following the general procedure A, the oxidation of **17**,**17'** afforded compound **18** as a white solid (68%). Following the general procedure C for 2 h with Wako MnO_2_, the oxidation of **17**,**17'** afforded compound **18** as a white solid (66%). ^1^H NMR (400 MHz, CDCl_3_) δ 8.26 (d, *J* = 2.2 Hz, 1H), 7.85 (d, *J* = 8.9 Hz, 1H), 7.70 (dd, *J* = 2.3, 8.9 Hz, 1H), 7.53–7.48 (m, 2H), 7.45–7.40 (m, 2H), 4.46–4.39 (m, 2H), 2.72 (t, *J* = 6.3 Hz, 2H), 2.03–1.97 (m, 2H); ^13^C NMR (100 MHz, CDCl_3_) δ 159.91, 156.71, 145.86, 138.58, 134.58, 132.71, 130.76, 130.24, 128.54, 123.91, 121.18, 119.46, 111.35, 67.21, 23.80, 21.75; IR (film): 3319, 3058, 2987, 2949, 2917, 2859, 1905, 1585, 1476, 1392, 1348, 1322, 1162, 1123, 1085, 989 cm^−1^; HRMS (ESI+, *m*/*z*): [M + H]^+^ calcd for C_18_H_14_BrClNO, 373.9942; found, 373.9933.

#### 3-(6-bromo-2-(4-chlorophenyl)quinolin-3-yl)propan-1-ol (19)

Following the general procedure B, the oxidation of **17**,**17'** afforded compound **19** as a white solid (60%). ^1^H NMR (400 MHz, CDCl_3_) δ 7.92–7.87 (m, 3H), 7.67 (dd, *J* = 2.2, 8.9 Hz, 1H), 7.44–7.37 (m, 4H), 3.51 (t, *J* = 6.2 Hz, 2H), 2.85–2.77 (m, 2H), 1.75–1.65 (m, 2H); ^13^C NMR (100 MHz, CDCl_3_) δ 159.9, 145.2, 139.0, 135.3, 134.8, 134.3, 132.8, 131.2, 130.3, 129.2, 128.9, 128.9, 120.8, 62.0, 33.3, 29.4; IR (film): 3353, 2924, 2847, 1783, 1732, 1598, 1476, 1431, 1393, 1258, 1188, 1085, 1059, 1009, 919, 823 cm^−1^; HRMS (ESI+, *m*/*z*): [M + H]^+^ calcd for C_18_H_16_BrClNO, 376.0098; found, 376.0090.

## Supporting Information

Supporting information features the characterization data of compounds **18**, **19**, **21** and **22,** copies of their ^1^H NMR and ^13^C NMR spectra, and the particle size analyses of MnO_2_ samples.

File 1 Experimental details.
