# Supplementary material for: Multicomponent reaction access to complex quinolines via oxidation of the Povarov adducts
Source: Beilstein J Org Chem. 2011 Jul 13;7:980–7. doi: 10.3762/bjoc.7.110 (PMC3170197; doi:10.3762/bjoc.7.110)
Supplement: File 1 — Experimental details. [file Beilstein_J_Org_Chem-07-980-s001.pdf]

Supporting Information

for

Multicomponent reaction access to complex quinolines via oxidation of the Povarov adducts

Esther Vicente-García[1], Rosario Ramón[1], Sara Preciado[1] and Rodolfo Lavilla*[1,2]

Address: [1]Barcelona Science Park, Baldiri Reixac 10–12, 08028, Barcelona, Spain and [2]Laboratory of Organic Chemistry, Faculty of Pharmacy, University of Barcelona, Avda. Joan XXIII sn, 08028, Barcelona, Spain

Email: Rodolfo Lavilla - rlavilla@pcb.ub.es

*Corresponding author

Experimental details

Table of contents

Synthetic procedures and characterization data for new compounds S2

^{1}H and ^{13}C NMR spectra S4

Particle size analyses of MnO_2 samples S6

5-(4-Chlorophenyl)-9-methyl-3,4-dihydrobenzo[h][1,6]naphthyridin-2(1*H*)-one (21a)

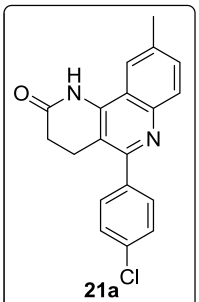

21a
$C_{19}H_{15}ClN_2O$
MW: 322,79

Following the general procedure A, the oxidation of **20a**,**20a'**, afforded compound **21a** as a white solid (22%).
Following the general procedure C for 5h with Wako MnO_2, the oxidation of **20a**,**20a'**, afforded compound **21a** as a white solid (71%). The spectroscopic data for this compound perfectly matches the published data [12].

Butyl-9-methoxy-5-[4-(trifluoromethyl)phenyl]-3,4 dihydrobenzo[h][1,6]naphthyridin-2(1*H*)-one (21b)

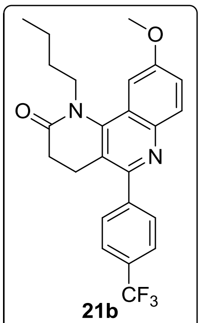

21b
$C_{24}H_{23}F_3N_2O_2$
MW: 428,45

Following the general procedure A, the oxidation of **20b**,**20b'**, afforded compound **21b** as a purple solid (84%).
Following the general procedure C for 8h with Wako MnO_2, the oxidation of **20b**,**20b'**, afforded compound **21b** as a purple solid (41%). The spectroscopic data for this compound perfectly matches the published data [12].

3-[2-(4-Chlorophenyl)-6-methylquinolin-3-yl]propanamide (22a)

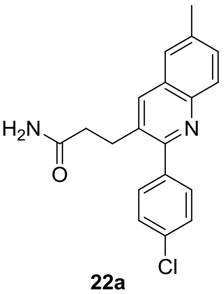

22a
$C_{19}H_{17}ClN_2O$
324,8041

Following the general procedure B, the treatment of **20a**,**20a'** afforded compound **22a** as a white solid (49%).
^{1}H NMR (400 MHz, $CDCl_3$) δ 8.00–7.93 (m, 2H), 7.55 (s, 1H), 7.51 (dd, *J*= 8.6, 1.9 Hz, 1H), 7.48–7.40 (m, 4H), 5.49 (br s, 1H), 5.39 (br s, 1H), 3.14–3.08 (m, 2H), 2.54 (s, 3H), 2.36–2.31 (m, 2H); **^{13}C NMR** (100 MHz, $CDCl_3$) δ 173.7, 158.1, 145.4, 139.2, 136.9, 135.9, 134.5, 131.9, 131.8, 130.3, 128.9, 128.8, 127.7, 126.0, 36.0, 28.5, 21.8 ppm. **IR** (film) ν_{max}: 3353, 3186, 3051, 2911, 2847, 1662, 1617, 1482, 1437, 1405, 1373, 1296, 1085, 1002, 918, 822, 133 cm^{-1}. **HRMS**: calcd for $C_{19}H_{18}ClN_2O$, 325.1102 ($M+H^+$); found, 325.1101.

3-[6-Methoxy-2-{4-(trifluoromethyl)phenyl}quinolin-3-yl]-*N*-pentylpropanamide (22b)

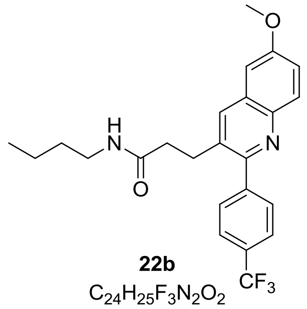

22b
$C_{24}H_{25}F_3N_2O_2$
MW: 430,46

Following the general procedure B, the treatment of **20b**,**20b'** afforded compound **22b** as a white solid (49%). The spectroscopic data for this compound perfectly matches the published data [12].

9-Bromo-5-(4-chlorophenyl)-3,4-dihydro-2H-pyrano[3,2-c]quinoline (18)

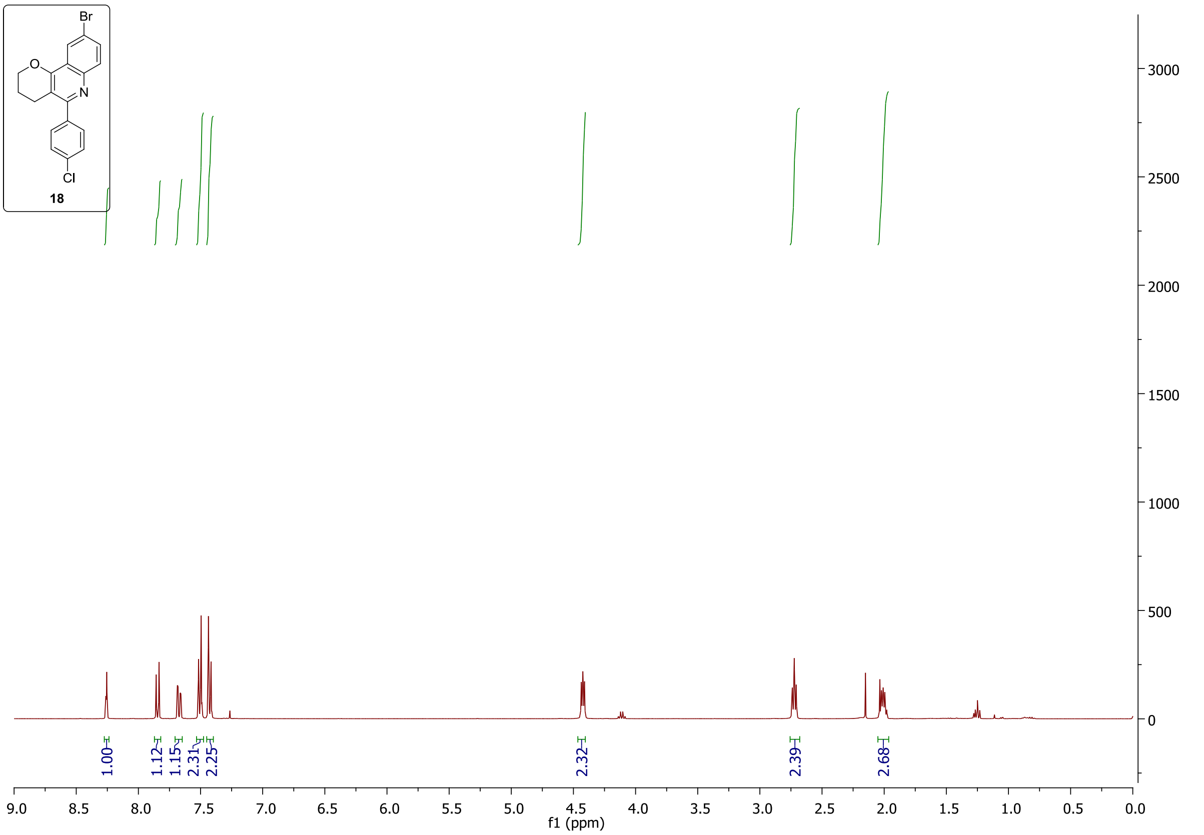

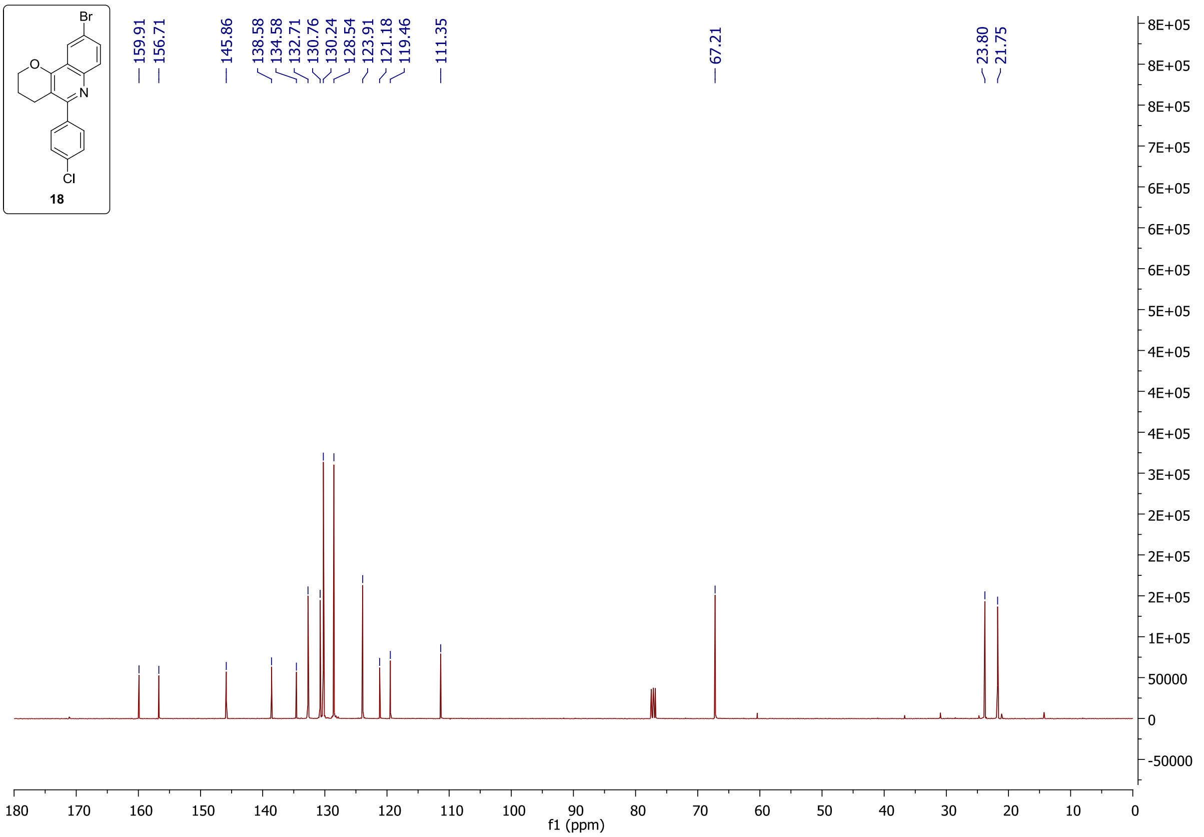

3-(6-Bromo-2-(4-chlorophenyl)quinolin-3-yl)propan-1-ol (19)

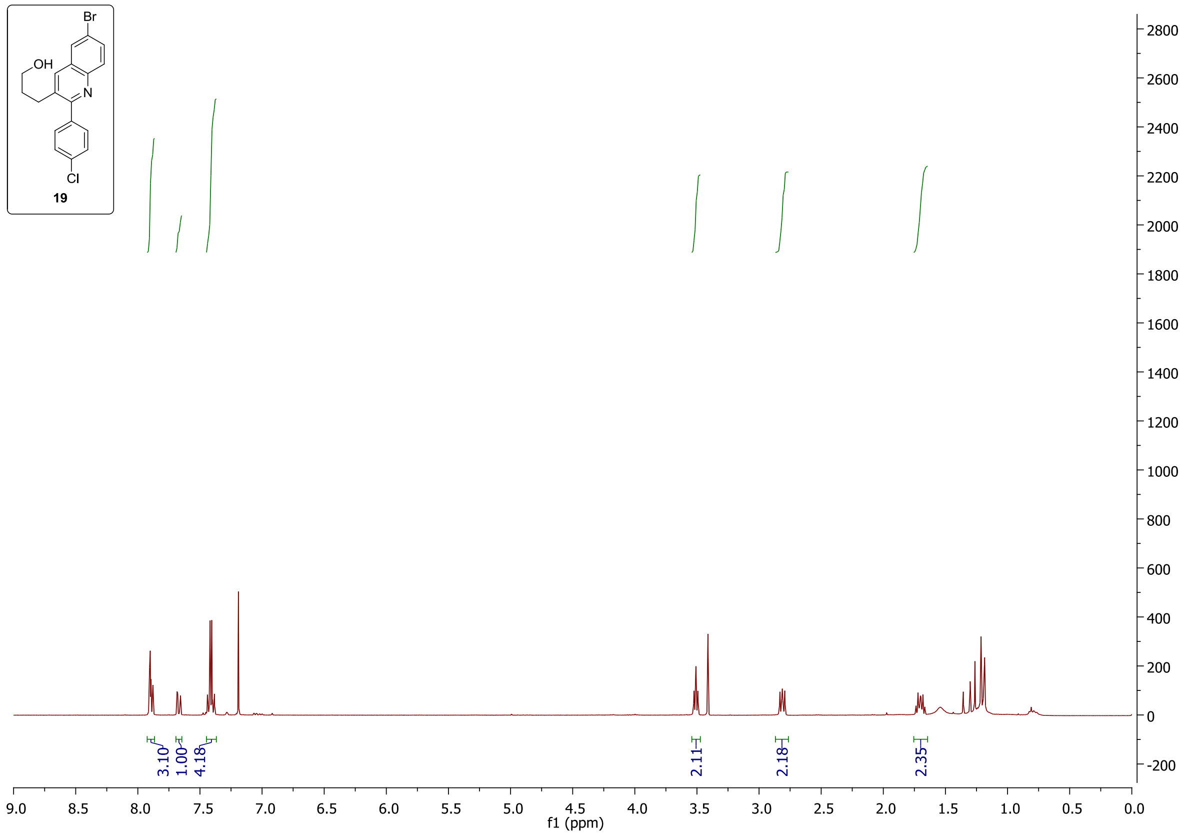

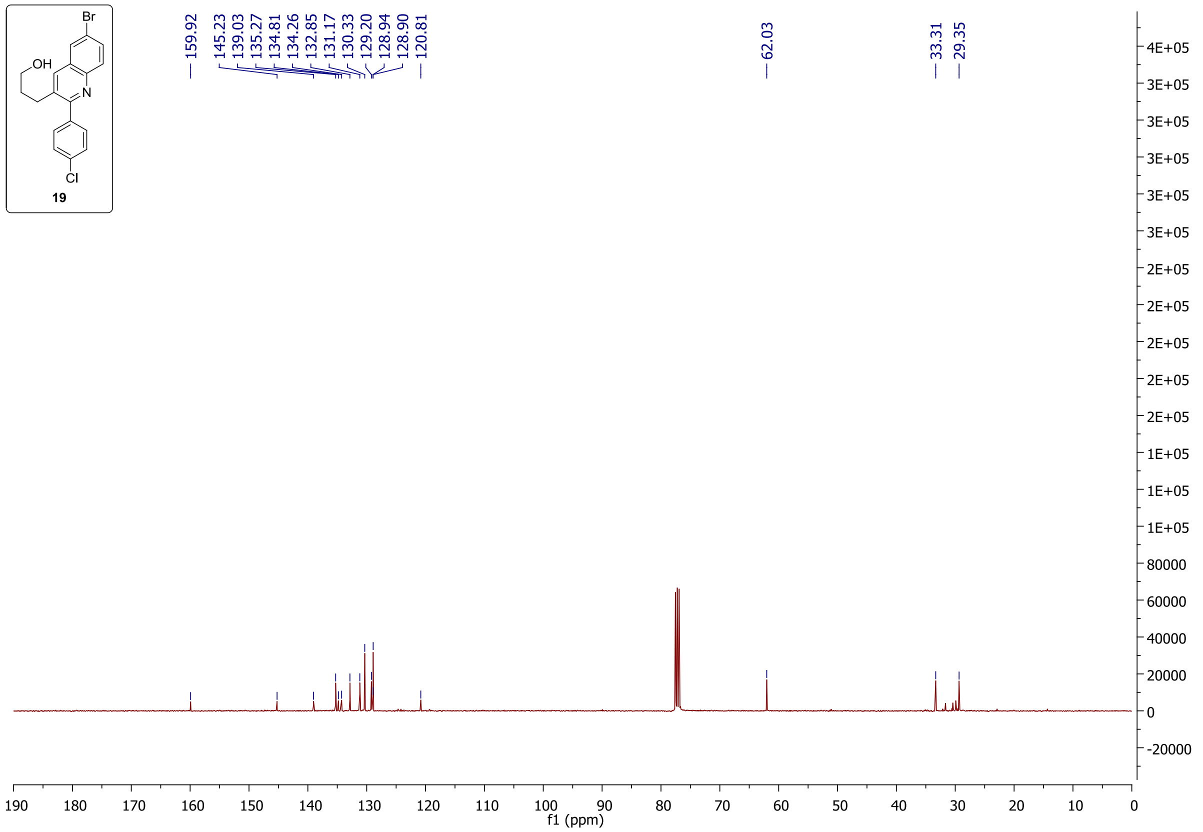

3-[2-(4-Chlorophenyl)-6-methylquinolin-3-yl]propanamide (22a)

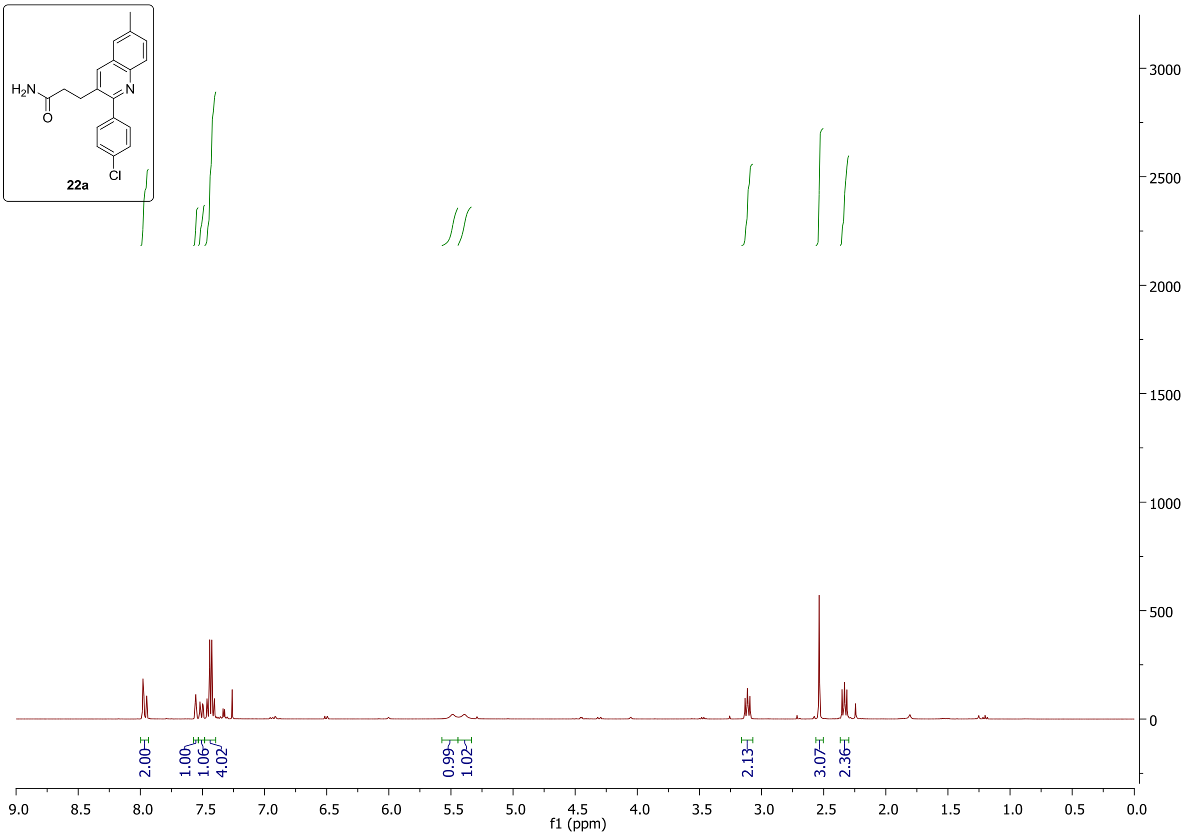

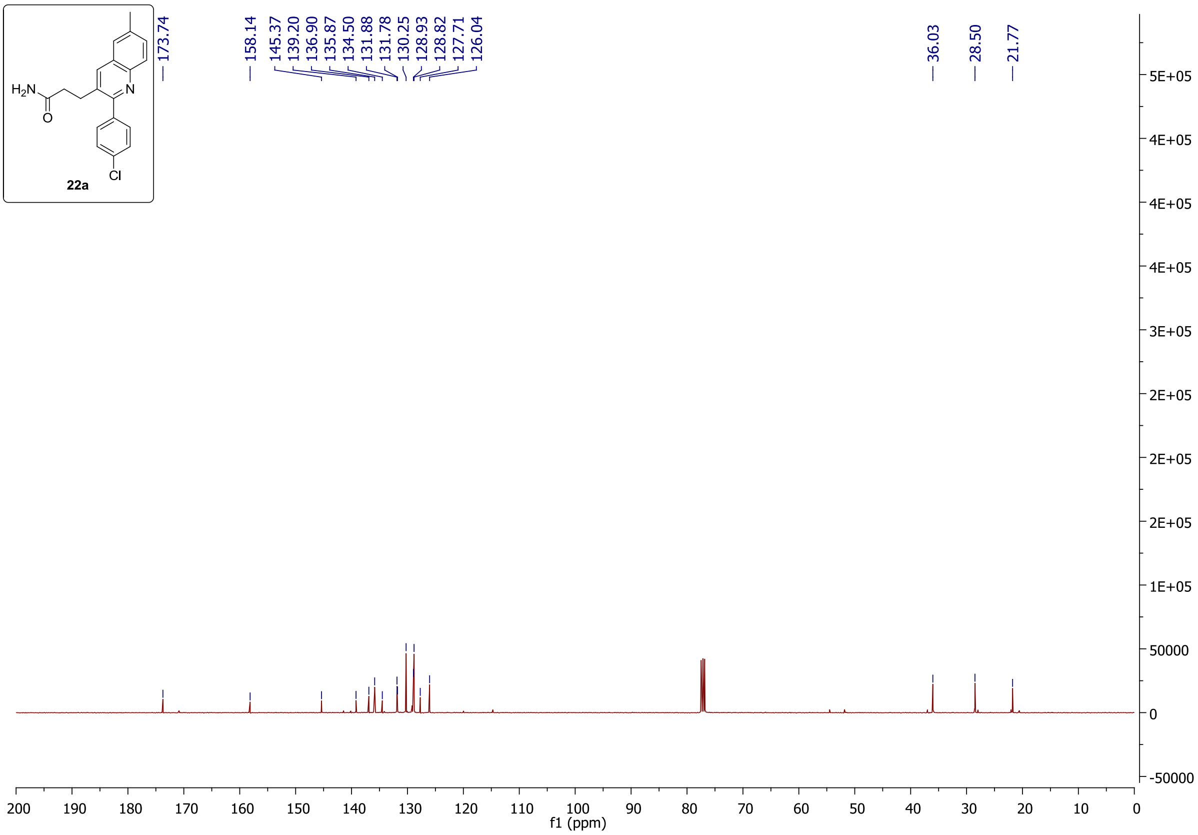

MnO_2 Particle size study:

All the manganese dioxide samples were analyzed with a LS™ 13 320 series Laser diffraction particle size analyzer to determine the particle size.

Reagent code	d_{10} (µm)	d_{50} (µm)	d_{90} (µm)	<75%	<95%	<10 µm (%)
310700	1.475	4.302	11.16	7.022	14.53	87.5
243442	77.66	138.4	173.2	156.8	183.2	1.57
217646	0.577	4.240	16.04	9.583	19.69	75.3
213490010	1.509	7.555	37.30	18.16	51.39	57.3
138-09675	2.743	25.70	63.10	44.77	73.05	21.6
Old Sample	0.932	11.46	47.78	24.40	66.57	47.7

d_{xx} (µm) indicates the size of particle below which XX% of the sample lies (50% - median diameter)

Aldrich (310700):

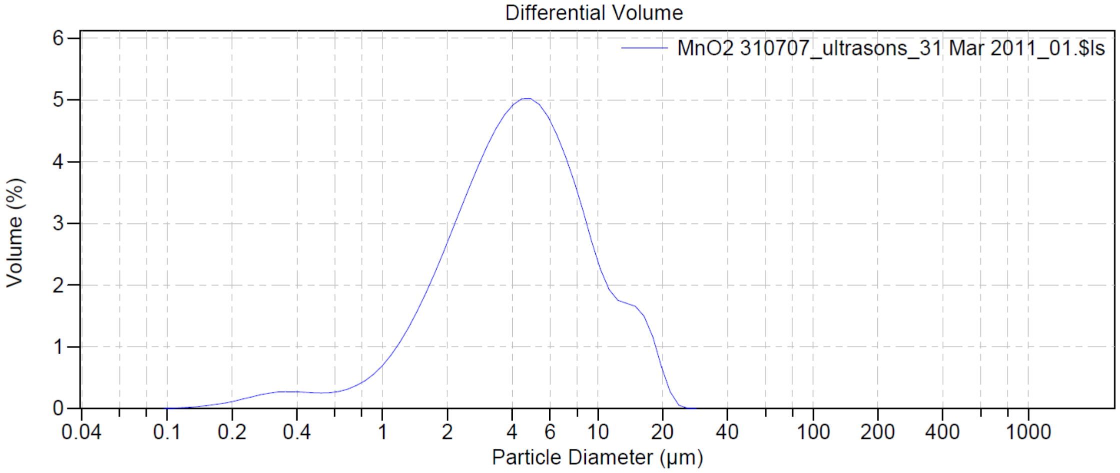

d_{10}: 1.475 µm d_{50}: 4.302 µm d_{90}: 11.16 µm

<75% 7.022 µm   <95% 14.53 µm

<10 µm 87.5%

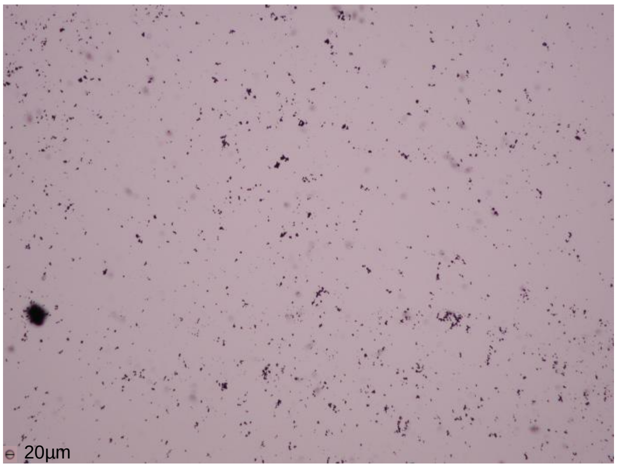

10x

Aldrich (243442):

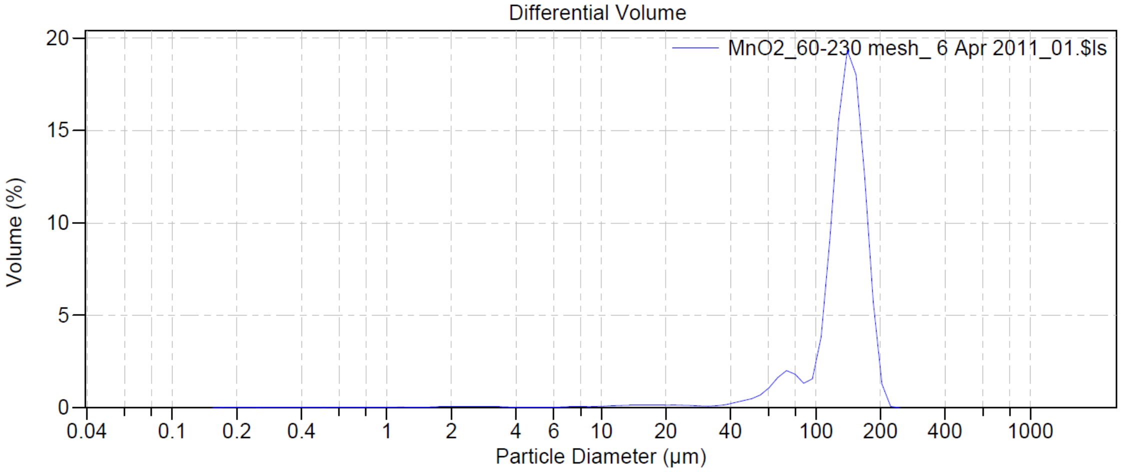

d_{10}: 77.66 μm $\underline{d_{50}\text{: 138.4 μm}}$ d_{90}: 173.2 μm

<75% 156.8 μm <u><95% 183.2 μm</u>

<10 μm 1.57%

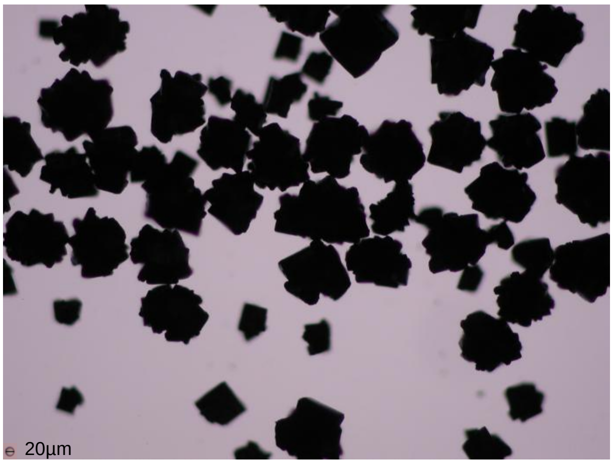

10x

Aldrich (217646):

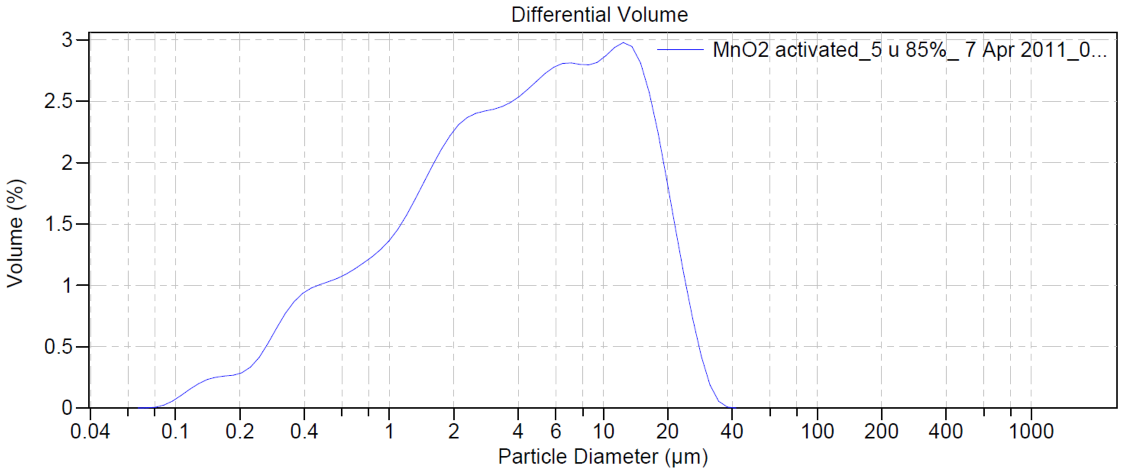

d_{10}: 0.577 µm $\underline{d_{50}\text{: 4.240 µm}}$ d_{90}: 16.04 µm

<75% 9.883 µm <u><95% 19.69 µm</u>

<10 µm 75.3%

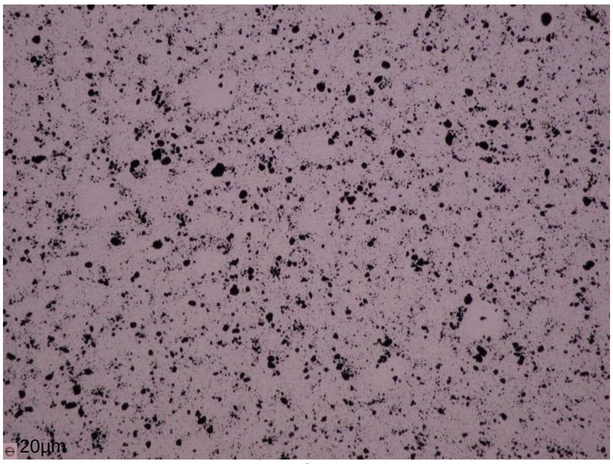

10x

Acros (213490010):

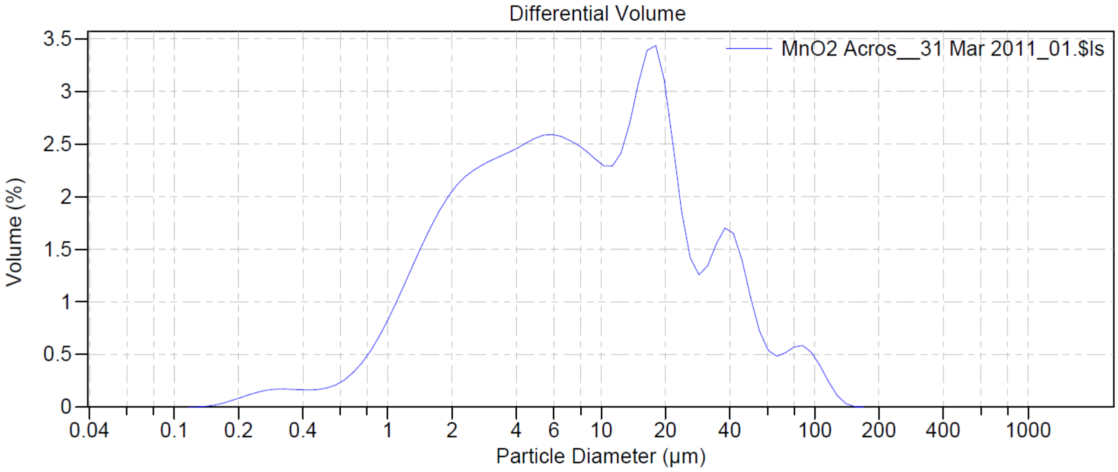

d_{10}: 1.509 µm d_{50}: 7.555 µm d_{90}: 37.30 µm

<75% 18.16 µm   <95% 51.39 µm

<10 µm 57.3%

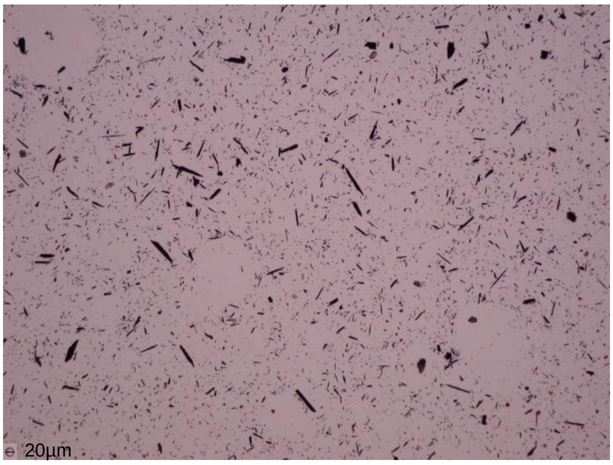

10x

Wako (138-09675):

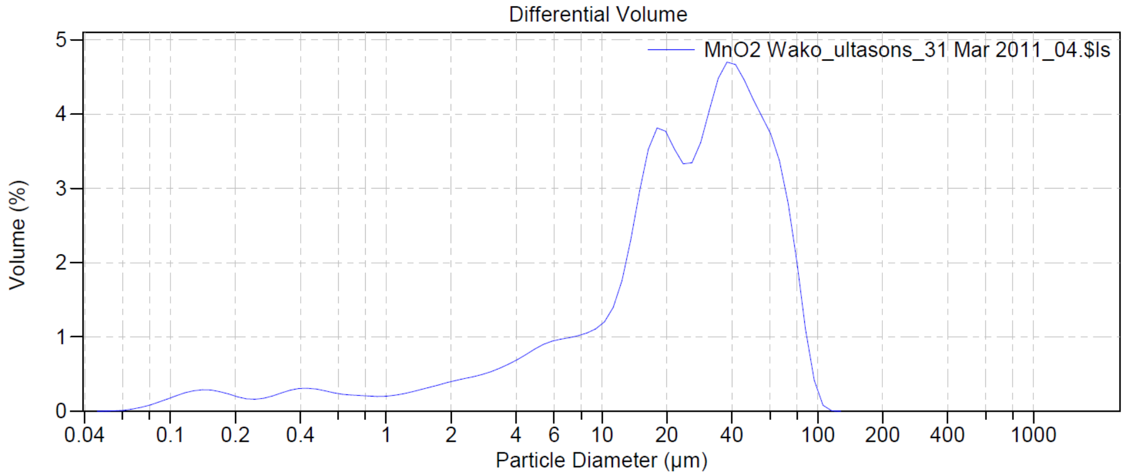

d_{10}: 2.743 µm d_{50}: 25.70 µm d_{90}: 63.10 µm

<75% 44.77 µm <95% 73.05 µm

<10 µm 21.6%

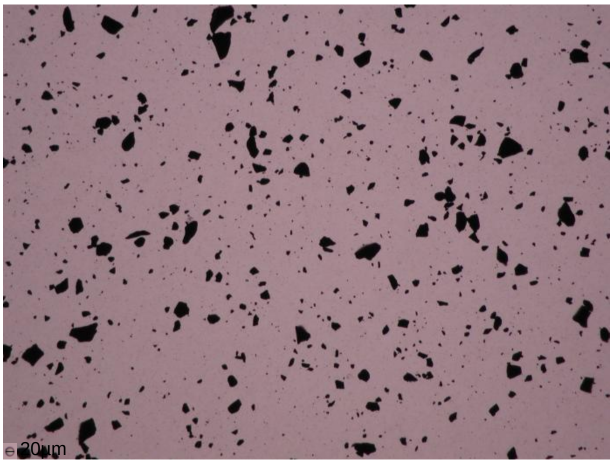

10x

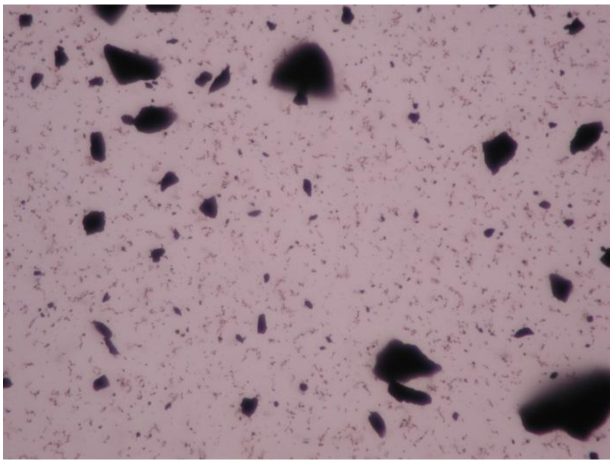

40x

MnO_2 Old Sample:

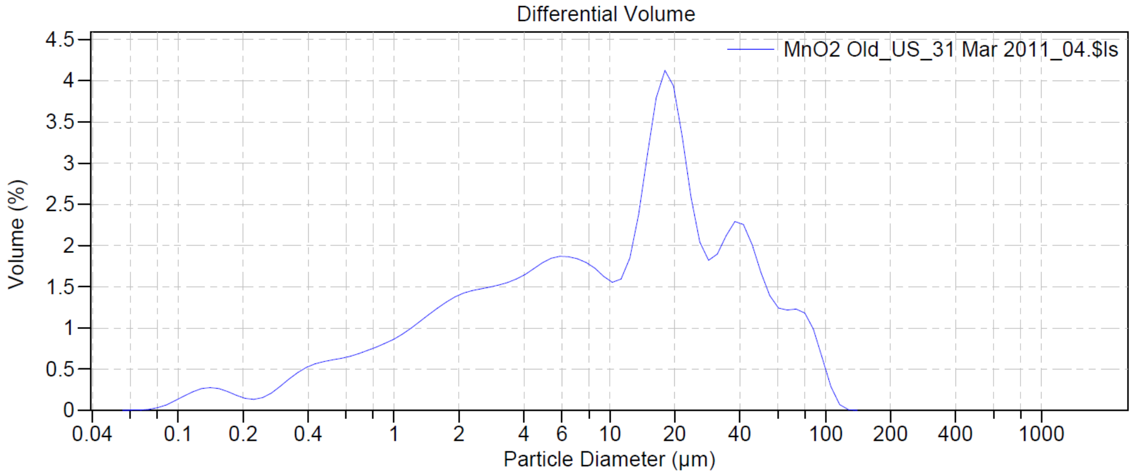

d_{10}: 0.932 µm d_{50}: 11.46 µm d_{90}: 47.78 µm

<75% 24.40 µm <95% 66.57 µm

<10 µm 47.7%

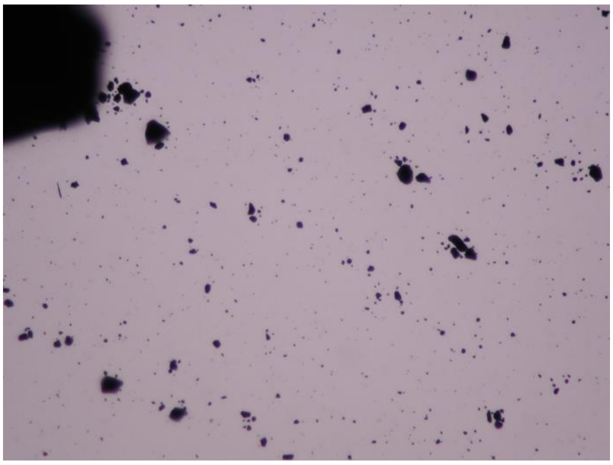

10x

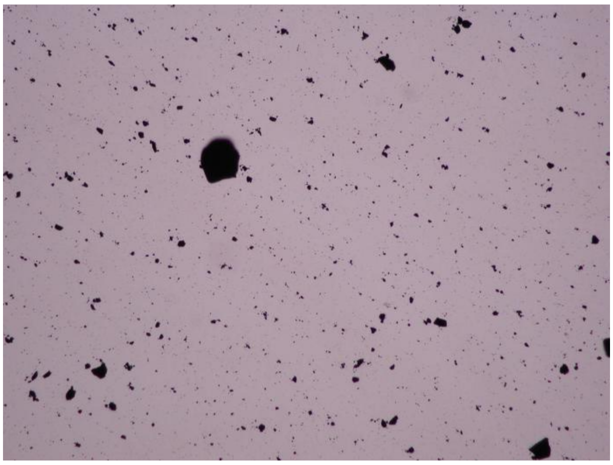

40x
